# The Effect of Adjuvant Chemotherapy on Survival in Patients with Residual Nasopharyngeal Carcinoma after Undergoing Concurrent Chemoradiotherapy

**DOI:** 10.1371/journal.pone.0120019

**Published:** 2015-03-23

**Authors:** Shiping Yang, Shaomin Lin, Qiang Fu, Baizhen Cai, Fei Kong, Guang Huang, Fafen Li, Han Wang

**Affiliations:** 1 Department of Radiation Oncology, Hainan Province People’s Hospital, Haikou, Hainan, PR China; 2 Department of Physiology, Hainan Medical college, Haikou, Hainan, PR China; H. Lee Moffitt Cancer Center & Research Institute, UNITED STATES

## Abstract

**Background:**

Guidelines from the U.S. National Comprehensive Cancer Network have recommended use of concurrent chemoradiotherapy (CCRT), followed by a 3-cycles combination of platinum and 5-fluorouracil chemotherapy as standard treatment for nasopharyngeal carcinoma (NPC). The benefits of CCRT for treatment of locally advanced NPC have been established. Whether platinum and 5-fluorouracil chemotherapy should be routinely added to locally advanced NPC after CCRT is still open to debate. Whether adjuvant chemotherapy provides an additional survival benefit for the subgroup of patients with residual nasopharyngeal carcinoma who have undergone CCRT is also unclear. This retrospective study was initiated to determine the survival benefit of adjuvant chemotherapy (AC) in residual NPC patients who have undergone concurrent chemoradiotherapy.

**Methods:**

The retrospective study included 155 nasopharyngeal carcinoma patients who had local residual lesions after the platinum-based CCRT without or with AC. Kaplan-Meier analysis and the log-rank test were used to estimate overall survival (OS), failure-free survival (FFS), local relapse-free survival (LRFS) and distant metastasis-free survival (DMFS).

**Results:**

Median follow-up was 47 months. Adjuvant cisplatin or nedaplatin plus 5-fluorouracil chemotherapy did not significantly improve 3-year OS, LRFS, FFS, and DMFS for patients with residual nasopharyngeal carcinoma after undergoing CCRT. The 3-year OS rates for the no-AC group and AC group were 71.6% and 73.7%, respectively (*P*= 0.44). The 3-year FFS rates for no-AC group and AC group were 57.5% and 66.9%, respectively ((*P*= 0.19). The 3-year LRFS rates for no-AC group and AC group were 84.7% and 87.9%, respectively ((*P*= 0.51). The 3-year DMFS rates for no-AC group and AC group were 71.4% and 77.4%, respectively ((*P*= 0.23).

**Conclusions:**

Since we did not find sufficient data to support significant survival in 3-year OS, LRFS, FFS, and DMFS, whether Adjuvant cisplatin or nedaplatin and 5-fluorouracil chemotherapy should be routinely added to residual nasopharyngeal carcinoma patients after undergoing CCRT remain uncertain.

## Background

Five studies have compared CCRT followed by adjuvant CT vs RT alone [1–5]. The first study was conducted by the Intergroup 0099 (IG 0099) and was reported by Al-Sarraf et al. in 1998 [6] and updated with 5-year results in 2001 [1] by the same authors.

Intergroup 0099 study showed that concurrent chemoradiotherapy with adjuvant chemotherapy showed a 31% increase in the 3 year overall survival [6]. Since 1998, this regimen has been strongly recommended for patients with advanced nasopharyngeal carcinoma. A major question regarding the design of the Intergroup 0099 regimen is the contribution of the adjuvant phase. There was three phase of pure adjuvant chemotherapy trials in nasopharyngeal carcinoma in which adjuvant chemotherapy was used alone [5, 7–8]. Therefore, it is unclear whether adjuvant chemotherapy provides any additional survival benefit over using concurrent chemoradiotherapy in advanced nasopharyngeal carcinoma.

A recent phase III randomized trial by Chen et al showed that adjuvant cisplatin and fluorouracil chemotherapy did not significantly improve FFS after CCRT in locoregionally advanced NPC. The trial also showed that the risk of treatment failure was not significantly decreased [9]. Does additional administration of chemotherapy after completing the CCRT phase not provide any additional benefit in all NPC patients? Or does it only benefit a few patients but not all of them?

Although NPC is very radiosensitive and chemoresponsive, in nearly 7%–13% of cases residual disease persists after treatment [10, 11]. The appearance of local or distant relapse can determines a less favorable prognosis for these patients [12]. In this regard, we defined a residual tumor as a tumor that did not regress completely after CCRT in 4 weeks. Is AC beneficial for these patients? Residual NPC was not mentioned in all the clinical trials of AC to a CCRT regime. Therefore it is unclear whether adjuvant chemotherapy provides an additional survival benefit in the subgroup of residual nasopharyngeal carcinoma patients who have undergone CCRT. Therefore, we retrospectively analyzed the outcomes of overall survival (OS), local relapse-free survival (LRFS), distant metastasis-free survival (DMFS) and failure-free survival (FFS) in a group with residual lesion of NPC patients who have undergone CCRT treated with or without AC. This study filled a gap in the knowledge of adjuvant chemotherapy treatment for the subgroup of patients with residual nasopharyngeal carcinoma who have undergone CCRT.

## Materials and Methods

We ensured that all the patients’ information was anonymized prior to beginning the analysis. This retrospective study was approved by the ethics committee of the Hainan Province People’s Hospital, Haikou, PR China. As written consent was waived, we gained oral consent which was obtained from the patients via telephone and conversations of this consent were recorded. The use of oral consent was approved by the Institutional Review Board. The study included 155 nasopharyngeal carcinoma patients with stage ~IVb, who had local residual lesions after the first course of cisplatin-based CCRT without or with AC (cisplatin or nedaplatin plus 5-fluorouracil) which was administered in our department from January 2006 to January 2011. 79 patients were treated with the concurrent chemoradiotherapy alone and 76 patients were treated with the concurrent chemoradiotherapy plus adjuvant chemotherapy. Bone scans, chest X-ray and abdominal ultrasound scans were also obtained from all patients with T2-T4 tumor or N1-N3 neck nodal disease. The inclusion criterion for this study was as follow: pathological confirmation for all primary tumors prior to CCRT; existence of local residual tumor after CCRT and absence of distant metastasis. Residual tumor was defined as residual disease by fibroendoscopy or MRI within 4 week after completion of CCRT. The criteria for residual disease on MRI were persistent tumor mass, thickened nasopharyngeal walls with enhancement at the primary site, or persistent enhancing lymph nodes that were present before CCRT. All patients were treated with 2.0–2.3 Gy per fraction with five daily fractions per week for 6–7 weeks. This was administered as megavoltage photons using either two-dimensional radiotherapy (2DRT), or three-dimensional conformal radio therapy (3DCRT). The cumulative radiation doses were 68 Gy or greater to the primary tumor and 60–66 Gy to the involved neck area. All potential sites of local infiltration and bilateral cervical lymphatic were irradiated to 50 Gy or greater. The concurrent chemo-radiotherapy consisted of cisplatin (100 mg/m^2^ every 21 d) for two or three cycles, followed (or not) by adjuvant cisplatin or nedaplatin (80 mg/m^2^ on day 1) and 5-fluorouracil (800 mg/m2 on days 1–5 every 3–4 wk). 24 patients had 2 cycles of adjuvant chemotherapy, and 52 patients had 3 cycles of adjuvant chemotherapy.

### Follow-up

All patients were followed up by our department at 3 months intervals for the first 2 years, every 6 months for 3–5 years, and annually thereafter. The median follow-up period for the whole group was 47 months (range, 8–93 months). All events were measured from the date of commencement of treatment. The following end points (time to the first defining event) were assessed: overall survival (OS), failure-free survival (FFS), local relapse-free survival (LRFS) and distant metastasis-free survival (DMFS). Local recurrence was established by fiberoptic endoscopy and biopsy and/or MRI. Distant metastases were diagnosed based on clinical symptoms, physical examination and imaging methods including chest X-ray, bone scan, CT/MRI scan and abdominal ultrasound.

### Statistical analysis

The differences in the distribution of selected demographic variables (age, gender, smoking status, alcohol status) and clinical characteristics (T stage, N stage, rT stage, rN stage, AC cycles and treatment) were evaluated using a x^2^ test. The actuarial rates of OS, LRFS, DMFS and FFS were calculated by the Kaplan-Meier method, and the differences were compared using the log-rank test. Multivariate analyses of prognostic variables for each outcome were performed using Cox proportional hazards modeling by a backward stepwise (Likelihood Ratio) procedure (Entry: 0.05; Removal: 0.10). Associations were quantified using hazard ratios and the 95% confidence intervals. Statistical significance was set at a *P*<0.05. All analyses were conducted using SPSS software (version 16.0.1; SPSS Inc., Chicago, IL).

## Results

### Patient characteristics

The baseline clinical characteristics are listed in Table 1. Medical and imaging records were retrospectively reviewed, and all patients before or after treating, then with CCRT were restaged according to the 7th edition of the AJCC [13]. Specifically with rTNM, “r” was defined as a residual tumor within 4 weeks after completion of CCRT, but not as a “recurrent tumor”. The two treatment groups were well balanced in terms of age, gender, cigarette smoking, drinking status, T classification, N classification, staging, and rT stage. The rN stage was different between the no-AC group and AC group, rN0 being slightly higher in the no-AC group (62% vs 48.7%) but not statistically significant.

**Table 1:** Characteristics of patients with residual tumor after completing CCRT.

	No adjuvant chemotherapy (n = 79)	Adjuvant chemotherapy (n = 76)	P*
Age (year)			0.971
≤45y	32(40.5%)	31(40.8%)	
>45y	47(59.5%)	45(59.2%)	
Gender			0.898
Male	62(78.5%)	59(77.6%)	
Female	17(21.5%)	17(22.4%)	
Cigarette smoking			0.864
Never	53(67.1%)	50(65.8%)	
Ever	26(32.9%)	26(34.2%)	
Drinking status			0.333
Yes	11(13.9%)	15(19.7%)	
No	68(86.1%)	61(80.3%)	
T classification			0.969
T2	24(30.4%)	22(28.9%)	
T3	40(50.6%)	40(52.7%)	
T4	15(19%)	14(18.4%)	
N classification			0.317
N1	27(34.2%)	35(46.1%)	
N2	40(50.6%)	31(40.8%)	
N3	12(15.2%)	10(13.1%)	
Staging			0.795
III	53(67.1%)	52(65.8%)	
IVa	15(19%)	16(21.1%)	
IVb	11(13.9%)	8(10.1%)	
rT stage			0.217
rT0	25(31.6%)	29(38.2%)	
rT1	15(19%)	18(23.7%)	
rT2	29(36.7%)	26(34.2%)	
rT3	10(12.7%)	3(3.9%)	
rN stage			0.232
rN0	49(62%)	37(48.7%)	
rN1	27(34.2%)	34(44.7%)	
rN2	3(3.8%)	5(6.6%)	
AC (cycles)			/
2 cycle	/	24(31.6%)	
3 cycle	/	52(68.4%)	

Abbreviations: CCRT = Concurrent Chemoradiotherapy; AC = Adjuvant chemotherapy.

*Two-sided x^2^ test.

### Survival

**Fig 1:**
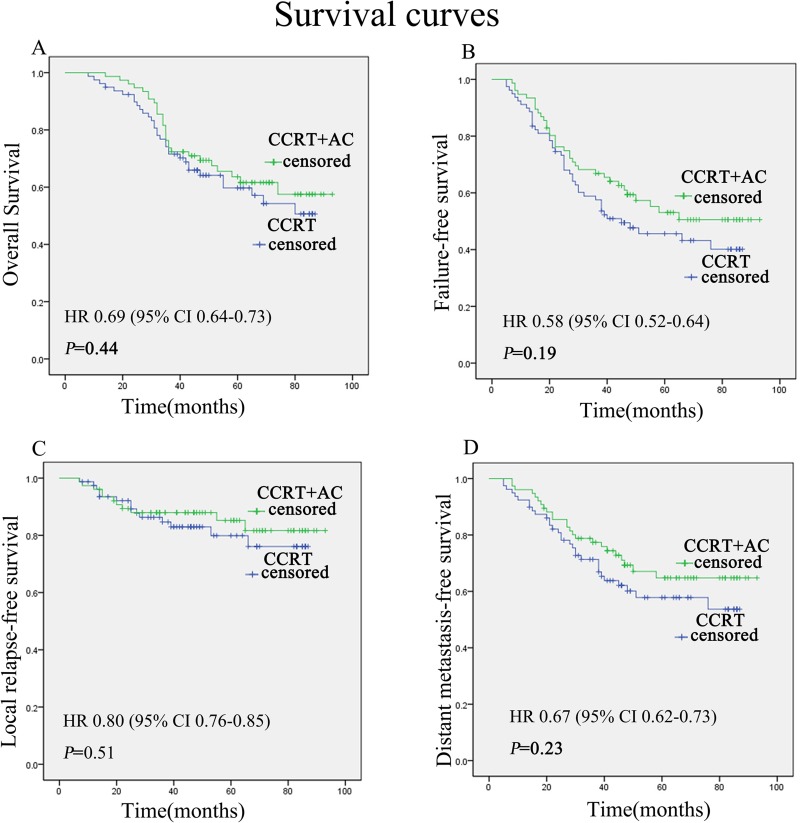
Kaplan-Meier survival curves for 155 patients with residual tumors after undergoing CCRT without or with AC. Overall survival (A), failure-free survival (B), local relapse-free survival (C), and distant metastasis-free survival (D). P values were calculated with the unadjusted log-rank test. CCRT = concurrent chemoradiotherapy; AC = adjuvant chemotherapy.

We found that using adjuvant cisplatin or nedaplatin and 5-fluorouracil chemotherapy did not significantly improve 3-year OS, LRFS, FFS, and DMFS for patients with residual nasopharyngeal carcinoma after undergoing CCRT. But it appears that there is a trend towards improvement in the outcome of adjuvant therapy group in the DMFS and FFS from the Kaplan-Meier curves. The 3-year OS rates for no-AC group and AC group were 71.6% and 73.7%, respectively (*P* = 0.44; HR = 0.69, 95% CI = 0.64–0.73; Fig. 1A). The 3-year FFS rates for no-AC group and AC group were 57.5% and 66.9%, respectively (*P* = 0.19; HR = 0.58, 95% CI = 0.52–0.64; Fig. 1B). The 3-year LRFS rates for no-AC group and AC group were 84.7% and 87.9%, respectively (*P* = 0.51; HR = 0.80, 95% CI = 0.76–0.85; Fig. 1C). The 3-year DMFS rates for no-AC group and AC group were 71.4% and 77.4%, respectively (*P* = 0.23; HR = 0.67, 95% CI = 0.62–0.73; Fig. 1D).

The seventy-eight patients with treatment failure are listed in Table 2. The no-AC group had forty-three patients that developed treatment failure. There was relapsing event in twelve patients and distant metastatic event in twenty-nine patients. And two patients had both distant metastasis and recurrence. Sixteen patients had developed distant metastasis in a single organ: six cases in bone, four cases in lung, five cases in liver and one case in mediastina lymph node. Thirteen patients had developed multi-organ metastasis. In AC group, thirty-five patients in all had developed treatment failure: there was relapsing event in eight patients, distant metastatic event in twenty-six patients and one patient had both distant metastasis and recurrence. Eleven patients had developed distant metastasis in a single organ: four cases in bone, two cases in lung and five cases in liver. Fifteen patients had developed multi-organ metastasis.

**Table 2:** Patterns of failure in the 155 patients after treatment.

Patterns of failure	CCRT	CCRT + AC
**Recurrence**	n (%)	n (%)
Primary recurrence	7 (8.9%)	5 (6.6%)
Nodal recurrence	5 (6.3%)	3 (3.9%)
**Distant metastasis**	n (%)	n (%)
Bone metastasis	6 (7.6%)	4 (5.3%)
Lung metastasis	4 (5.1%)	2 (2.6%)
Liver metastasis	5 (6.3%)	5 (6.6%)
Mediastina metastasis	1 (1.3%)	0 (0.0%)
Multiple metastasis	13 (16.5%)	15 (19.7%)
**Distant metastasis, Primary and/or nodal recurrence**	2 (2.5%)	1 (1.3%)

Abbreviation: CCRT = concurrent chemoradiotherapy; CCRT + AC = concurrent chemoradiotherapy + adjuvant chemotherapy.

### Prognostic factors

To identify the factors which affected patient outcome, we performed multivariate analyses to evaluate the prognostic value of age, gender, cigarette smoking, alcohol use, T stage, N stage, rT classification, rN classification and AC cycles (Table 3).

**Table 3:** Effect of prognostic factors on survival in multivariable analyses.

	OS		LRFS		DMFS		FFS	
	HR(95%CI)	*P* *	HR(95% CI)	*P* *	HR(95% CI)	*P* *	HR(95% CI)	*P* *
Age								
≥45y vs <45 y	0.70(0.41–1.19)	0.19	0.72(0.32–1.64)	0.43	0.98(0.55–1.75)	0.94	0.94 (0.58–1.52)	0.79
Gender								
Female vs Male	0.99(0.54–1.81)	0.98	0.72(0.30–1.73)	0.46	1.73(0.82–3.69)	0.15	1.50(0.82–2.72)	0.19
Smoking status								
Ever vs N ever	0.60(0.32–1.10)	0.97	0.61(0.33–1.12)	0.11	0.66(0.26–1.69)	0.39	1.29(0.68–2.46)	0.44
Drinking status								
Ever vs Never	0.82(0.44–1.54)	0.53	0.60(0.32–1.03)	0.10	0.73(0.25–2.04)	0.54	1.47(0.78–2.80)	0.23
T classification								
T3–T4 vs T2	1.35(0.77–2.37)	0.29	3.24(1.08–9.67)	0.04	1.15(0.65–2.02)	0.64	1.50(0.91–2.47)	0.11
N classification								
N2–N3 vs N1	1.75(0.99–3.08)	0.05	1.22(0.53–2.81)	0.64	2.02(1.10–3.73)	0.02	1.74(1.06–2.87)	0.03
rT classification								
rT1–3 vs rT0	0.88(0.35–2.20)	0.79	0.52(0.10–2.61)	0.43	1.25(0.54–2.89)	0.61	0.97(0.46–2.03)	0.94
rN classification								
rN1–2 vs rN0	0.90(0.36–2.25)	0.82	0.60(0.11–2.76)	0.48	1.41(0.61–3.29)	0.43	1.14(0.54–2.41)	0.72
Treatment group								
CCRT vs CCRT + AC	0.87(0.51–1.47)	0.59	0.76(0.33–1.75)	0.52	0.71(0.41–1.24)	0.23	0.73(0.45–1.16)	0.18
AC cycles								
2-cycle vs 3-cycle	0.79(0.34–1.84)	0.59	0.78(0.20–2.99)	0.78	0.84(0.34–2.10)	0.71	0.84(0.39–1.81)	0.66

* Age, gender, cigarette smoking, alcohol use, T stage, N stage, rT stage, rN stage, AC cycles and treatment method in multivariate Cox mode.

On multivariable analysis, treatment method was not a significant predictive factor for OS, LRFS, DMFS, or FFS (Table 3). Advanced T stage had independent poor prognostic factors for LRFS. Advanced N stage had independent poor prognostic factors for DMFS and FFS. The results showed that compared to patients with T2 stage NPC, patients with T3–T4 stage had an approximately 3-fold increased risk of local relapse (HR = 3.24; 95% CI, 1.08–9.67; *P* = 0.04; Table 3). Patients with N1 stage NPC, patients with N2–N3 stage had an approximately 2-fold increased risk of distant metastasis (HR = 2.02; 95% CI, 1.10–3.73; *P* = 0.02; Table 3). There was no significant association between the remaining predictors (age, gender, smoking status, alcohol use, rT stage, rN stage and AC cycles) and the survival, recurrence or metastasis rates.

## Discussion

Local failure can be divided in residual or recurrent disease. The definition of residual disease is arbitrary. Usually it is defined as the confirmation of disease occurring within 6 months after treatment [11, 14–16]. In our report, the definition of residual NPC is residual tumor within 4 week of completion of primary chemoradiotherapy. We aim to evaluate the therapeutic benefit of AC modality for the subgroup of patients with residual nasopharyngeal carcinoma who have undergone CCRT.

### Comparison of survival benefit

Survival after treatment for NPC depends on the stage of the disease, chemotherapy regimen, irradiation technique, doses delivered and the socio-economic conditions [17]. Recently published randomized clinical studies have demonstrated a 71–88% 3-year OS rates and 54–88% 3-year FFS rates in the CCRT arm [2–4], while our study showed a 71.6% 3-year OS and 57.5% 3-year FFS the CCRT arm.

There are several possible reasons for the differences. First, all our patients were III and IV disease in our study. Chua et al. demonstrated that the 5 year OS rates in the 1997 AJCC Stage I, II, III, and IV disease were 97.7%, 78.7%, 79.5% and 61.4%, respectively [18]. The proportion of 100% III and IV disease, indicated that our patients with unfavorable prognostic factors. Second, all RT in our study was based on the 2D or 3D technique with conventional fractionation. The advantages of IMRT over conventional radiation therapy include local-regional control and improved survival rates and quality of life in NPC patients [19].

The effect of adjuvant chemotherapy has not been reported in the English literature for patients with residual nasopharyngeal carcinoma. We initiated this study to rectify that situation and to provide an English language study on this subject.

### The possible explanation for our result

The optimal length of the interval to retreatment of NPC patients with residual tumor is a topic of debate. Some investigators advocate treating as soon as possible in order to prevent progression and dissemination, while others advocate waiting for at least 10 weeks, because late histological regressions might occur [16, 20]. Our retrospective study shows that adjuvant chemotherapy provides no additional survival benefit over CCRT in the subgroup of patients with residual nasopharyngeal carcinoma. The results might be connected with the spontaneous regression of late-responding tumors. Kwong et al [20] studied the histological regression and performed serial post treatment-nasopharyngeal biopsies in 803 patients. 70% of the post-radiation positive biopsies regressed within 12 weeks without additional treatment. There was no difference between the late and early regression in 5-year local control rate, regional and distant metastasis-free rate and overall survival. A high proportion of early positive histology remitted spontaneously. Delayed histologic remission in NPC patients is not a poor prognostic factor and additional treatment is not necessary. We can derive that retreatment of residual NPC who have undergone CCRT is not necessary as soon as possible because of the spontaneous regression of late-responding tumors. Our study shows the patients with residual NPC had no benefit from additional chemotherapy before the fourth week of post-chemoradiation. This justifies a wait-and-see policy for residual NPC until the tenth week.

### Limitations of this study

Firstly, treatment variability might be one of the limitations in this study. This is due to limited medical resources, as 2DRT and 3DCRT were used instead of IMRT. Secondly, although most patients with AC in this study received a scheme of cisplatin and 5-fluorouracil, there were some patients with AC who received a scheme of nedaplatin and 5-fluorouracil. Furthermore, our study was a retrospective study, and the conclusions need to be confirmed by future prospective studies.

We did not find sufficient data in support of the benefit of 3-year OS and LRFS for the adjuvant chemotherapy group. The addition of adjuvant chemotherapy to CCRT in patients with local residual lesions does not significantly improve survival rates in 3-year DMFS and DFS. But it appears that there is a trend towards improvement in the outcome of the adjuvant therapy group in DMFS and DFS from the Kaplan-Meier curves. On account of the fact that this study is retrospective, a number of factors concerning patients and tumor characterstics could not be controlled. Furthermore, the relatively small number of patients is also a limiting factor for this study. It is essential that large randomized clinical trial (RCT) with long follow-up periods be conducted to properly evaluate any significant survival benefit that might occur for patients with residual nasopharyngeal carcinoma after undergoing CCRT from the addition of AC.

## Conclusions

This study is the first attempt to evaluate the prognostic value of AC in residual NPC after CCRT. Our analysis demonstrates that adjuvant cisplatin or nedaplatin and 5-fluorouracil chemotherapy did not significantly improve 3-year OS, LRFS, FFS, and DMFS survival rates for patients with residual nasopharyngeal carcinoma after undergoing CCRT. Long follow-up periods need to be conducted to properly evaluate survival benefit. Whether AC should be routinely added to residual nasopharyngeal carcinoma after CCRT is remain uncertain. Therefore, AC should not be used routinely except for a clinical trial.
